# Synthesis of Novel IP Agonists via *N*-Aminoethyl Cyclic Amines Prepared by Decarboxylative Ring-Opening Reactions

**DOI:** 10.3390/molecules17021233

**Published:** 2012-01-31

**Authors:** Yasuhiro Morita, Takeshi Ishigaki, Kuniaki Kawamura, Ryoji Hayashi, Masafumi Isogaya, Mika Kitsukawa, Mitsuko Miyamoto, Masashi Uchida, Katsuhiko Iseki

**Affiliations:** Pharmaceutical Research Laboratories, Toray Industries Inc., 6-10-1 Tebiro, Kamakura, Kanagawa 248-8555, Japan

**Keywords:** amines, anilines, piperidines, heterocycles, IP agonists

## Abstract

An efficient synthesis of a highly potent and selective IP (PGI_2_ receptor) agonist that is not structurally analogous to PGI_2_ is described. This synthesis is accomplished through the following key steps: Nucleophilic ring-opening of 3-(4-chlorophenyl)-oxazolidin-2-one prepared by a one-pot procedure with 4-piperidinol and selective *O*-alkylation of 1-(2-(4-chlorophenylamino)ethyl)piperidin-4-ol. The obtained compound is a potent and selective IP agonist displaying a long duration of action.

## 1. Introduction

**Figure 1:**
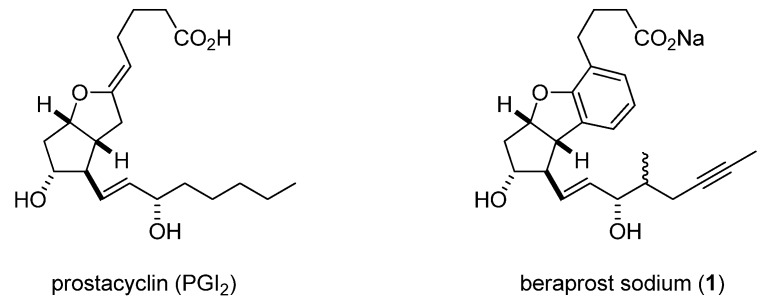
Chemical structures of prostacyclin (PGI_2_) and beraprost sodium (**1**).

Prostacyclin (PGI_2_; Figure 1) is an endogenous IP agonist generated in vascular endothelial cells, that has potent inhibitory effects on platelet adhesion and aggregation, as well as on vasoconstriction [1]. However, the therapeutic application of PGI_2_ itself is severely restricted by its chemical instability due to its chemically labile enol ether moiety. To date, orally active prostacyclin analogues [2,3,4,5] with improved stability of the enol ether moiety have been reported. Among them, beraprost sodium (**1**; Figure 1), which has a modified omega-side chain and a phenyl ether instead of an enol ether, is chemically and metabolically stable and hence was the first orally active prostacyclin analogue discovered by Toray Industries, Inc. [6]. This stable PGI_2_ analogue has been used to treat chronic occlusive disease since 1992 and primary pulmonary hypertension since 1999.

**Figure 2:**
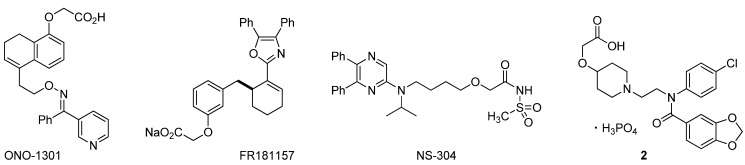
Chemical structure of non-prostanoid PGI_2_ mimetics.

Beraprost sodium shows a wide variety of clinically useful pharmacological effects, such as antiplatelet effects [7,8], vasodilatory effects [9], inhibition of inflammatory cytokine production [10], and inhibition of proliferation of vascular smooth muscle cells [11], but also has some drawbacks including short duration of action. To overcome these drawbacks, some potent non-prostanoid IP agonists (Figure 2) were recently reported and provided a possible solution [12,13,14,15,16,17,18,19].

In our research efforts seeking new IP agonists superior to beraprost sodium, we have identified 2-((1-(2-(*N*-(4-chlorophenyl)benzo[*d*] [1,3] dioxole-5-carboxamido)ethyl)piperidin-4-yl)oxy)acetic acid phosphoric acid salt (**2**) [20], which inhibits platelet aggregation and is expected to have improved pharmacokinetic properties, including longer plasma half-life compared with that of beraprost sodium. Early in the search for novel IP agonists, complicated multistep sequences were used to prepare designed molecules, including **2**, but to make strategic progress in drug discovery programs, an efficient synthesis of multigram quantities of **2** is indispensable. Against this background, we have now developed a short and practical method for synthesizing *N*-aminoethyl cyclic amines by decarboxylative ring-opening of *N*-aryloxazolidin-2-ones [21,22]. Herein, we report an efficient synthesis of **2** by this newly developed ring-opening reaction.

## 2. Results and Discussion

### 2.1. Retrosynthetic Analysis of ***2***

**Scheme 1:**
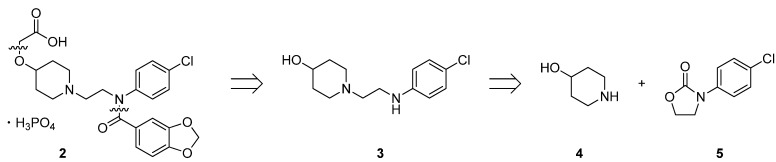
Synthetic strategy.

Our retrosynthetic analysis is outlined in Scheme 1. The target molecule **2** would be synthesized via *O*-alkylation and *N*-amidation of 1-(2-(4-chlorophenylamino)ethyl)piperidin-4-ol (**3**). We envisaged that key intermediate **3** could be constructed by decarboxylative ring-opening of 3-(4-chlorophenyl)-oxazolidin-2-one (**5**) with 4-piperidinol (**4**). Accordingly, we started our synthetic studies by seeking a general and practical method for preparing *N*-aminoethyl cyclic amines, including **3**.

### 2.2. Decarboxylative Ring-Opening Reaction

**Figure 3:**
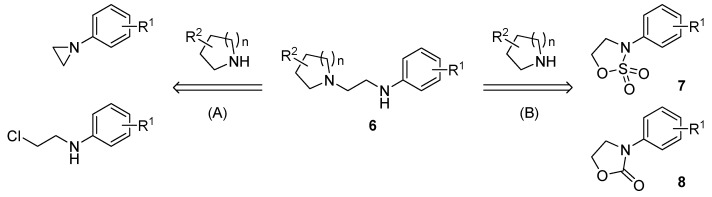
Possible synthetic routes to *N*-aminoethyl cyclic amines having scaffold **6**.

On the basis of preceding work [23,24,25,26,27,28], we planned to prepare **6** by two synthetic routes (Figure 3). Route A is direct and simple, involving the reaction of an amine with a substituted aziridine or its precursor (e.g., *N*-substituted chloroethylamine) [23]. However, aziridines are not commercially available at present because of their toxic and carcinogenic properties [29,30], and thus we synthesized *N*-substituted chloroethylamines by a well-known route [31,32]. Although this route is attractive for preparing various substrates, multiple steps including a reduction step are required. The other route is the ring-opening reaction of a cyclic sulfamidates **7** or *N*-aryloxazolidin-2-ones **8** with a secondary amine (Figure 3, route B). Generally, primary and secondary amines react effectively with five-membered cyclic sulfamidates under mild reaction conditions to furnish the corresponding diamines in good yield.However, the utility of **7** as a substrate is limited since their preparation requires multiple steps, including oxidation [24]. This method using **8** has been previously reported by Poindexter and co-workers [25,26,27].

**Scheme 2:**
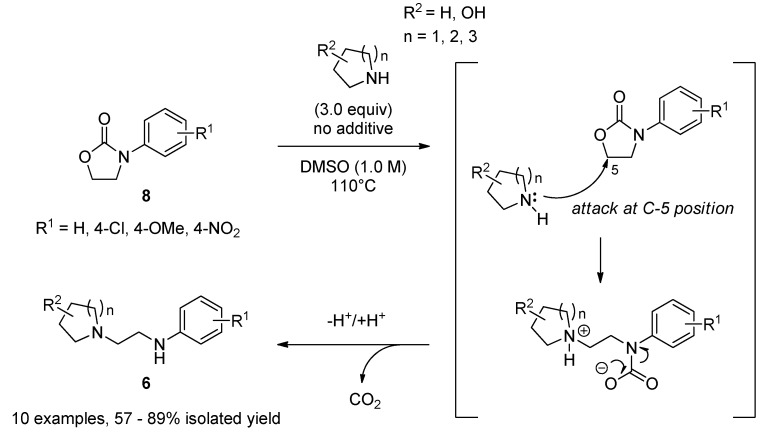
Decarboxylative ring-opening of **8**, and plausible reaction mechanism.

This previous work makes clear that an improved decarboxylative ring-opening reaction would lead to a more direct synthesis of **6**. However, an initial attempt using **5** (**8**, R^1^ = 4-Cl) and 4-piperidinol hydrogen chloride salt yielded the desired product **3** in low HPLC yield (3%) using a modification (in DMSO; 100 °C, 24 h) of the conditions reported by Poindexter and co-workers [21,22]; therefore, we conducted extensive screening of amines (salt and/or free), solvents, and temperatures in order to improve the yield. We found that nucleophilic ring-opening using **8** with free cyclic amines proceeded under heating in DMSO. Furthermore, this reaction under the optimized conditions had a wide substrate scope (R^1^, R^2^, and n shown in Scheme 2), and furnished *N*-aminoethyl cyclic amines in moderate yield: 57–89% for 10 examples (Scheme 2) [22].

As mentioned above, we have successfully developed an efficient conversion of *N*-aryloxazolidin-2-ones **8** to *N*-aminoethyl cyclic amines **6** [22]. This method can be applied to the synthesis of novel IP agonists.

### 2.3. Synthesis of ***2***

**Scheme 3:**
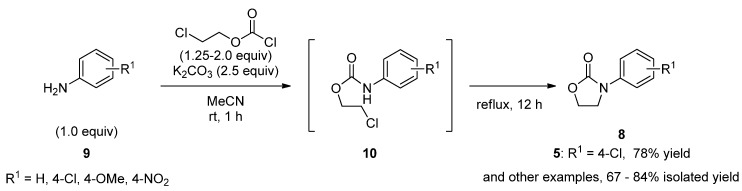
One-pot preparation of **8**.

Toward the total synthesis of target molecule **2** starting from **9**, we first attempted to synthesize **5** to be used as substrate in the decarboxylative ring-opening reaction. Several methods for preparing **8** are known [33,34,35,36,37], and one typical and efficient method consisting of two steps: first, formation of *N-*aryl-2-chloroethyl carbamate from an aniline and 2-chloroethyl chloroformate in the presence of an organic base such as tertiary amine or an inorganic base such as sodium, potassium, or calcium carbonate; and second, strong-base-induced intramolecular cyclization of the isolated carbamate. While both weak and strong bases have been employed for *N*-acylation and intramolecular cyclization in this method, we attempted to improve the two-step reaction sequence to a one-pot method using one kind of base. In this study, we found the practical one-pot conditions shown in Scheme 3. Thus, aniline **9** was reacted with 2-chloroethyl chloroformate (1.25–2.0 equiv.) in the presence of potassium carbonate (2.5 equiv.) in acetonitrile at room temperature to give 2-chloroethyl substituted phenylcarbamate **10** *in situ*, which was then heated at reflux to induce intramolecular cyclization, affording the desired product **8** in satisfactory yield.

We next focused on the decarboxylative ring-opening of **5** toward **3**. According to the protocol developed in our previous report (Scheme 2) [21,22], decarboxylative ring-opening of 1 mmol of **5** in a typical example with free amine 4-piperidinol (**4**) under heating in DMSO (1.0 M) gives **3** without any side reactions. In our ongoing studies, the required reaction time was investigated over a range of reaction temperatures (70 to 150 °C). The results of the temperature screening are summarized in Table 1. Compound **3** was obtained in good HPLC yield (>80%) at high reaction temperatures (>110 °C; entries 3, 4, 5). Under the reaction conditions of entry 3, we continued the reaction until **5** was completely consumed, as determined by HPLC analysis. The reaction took 5 days to reach completion, giving the desired product **3** in 81% isolated yield (99.0% purity by HPLC peak area).

**Table 1:** Optimization of reaction conditions *^a^*. 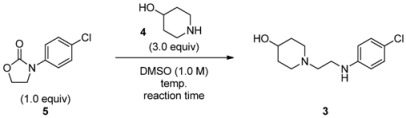

Entry	Temp. (°C)	Yield (%) of 3 *^b^* at reaction time		
		1 day	2 days	3 days
1	70	17	27	34
2	90	46	61	69
3	110	81	92	95
4	130	96	96	96
5	150	95	98	99

*^a^* Using *N*-(*p*-chlorophenyl) oxazolidin-2-one **5** (1 mmol); *^b^* HPLC yield by peak area at 254 nm: 100 × **3**/[**3**+**5**].

Next, we turned our attention to the construction of the oxyacetic acid and piperonyl amide moieties. In our initial studies, we carried out *N*-acylation with piperonyloyl chloride. This reaction, however, produced several compounds including desired product **11**, *N-* and *O*-acylated product **12**, and decomposition products (Table 2).

**Table 2:** Construction of the piperonyl amide moiety *^a^*. 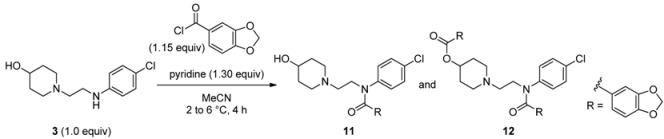

Compounds	Yield (%) *^b^*
**11**	69.4
**12**	20.5
Piperonic anhydride	7.7
**3**	1.2
Piperonic acid	0.3

*^a^* Using 1-(2-((4-chlorophenyl)amino)ethyl)piperidin-4-ol (**3**) (99.7% HPLC purity); *^b^* HPLC yield after workup, by peak area at 210 nm: 100 × (compound peak/total peak area).

**Scheme 4:**
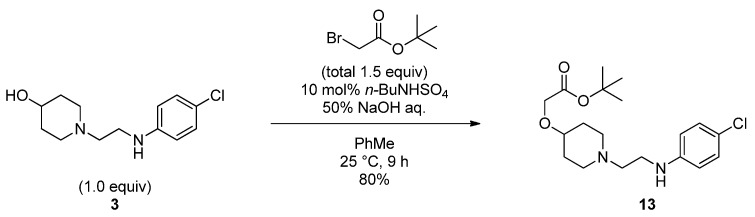
*O*-Alkylation of **3** under biphasic conditions.

Since our initial attempt resulted in lower selectivity for introducing the piperonyl group to the aniline nitrogen atom of **3** because of the low nucleophilicity of the nitrogen atom, we next focused on the following synthetic sequence: *O*-Alkylation and then *N*-acylation. After thoroughly screening conditions for *O*-alkylation, we found that by using a phase transfer catalyst in a biphasic mixture of toluene and 50% NaOH aqueous solution, the reaction proceeded smoothly to afford the desired product **13** with no *N*-alkylated product (Scheme 4).

**Scheme 5:**
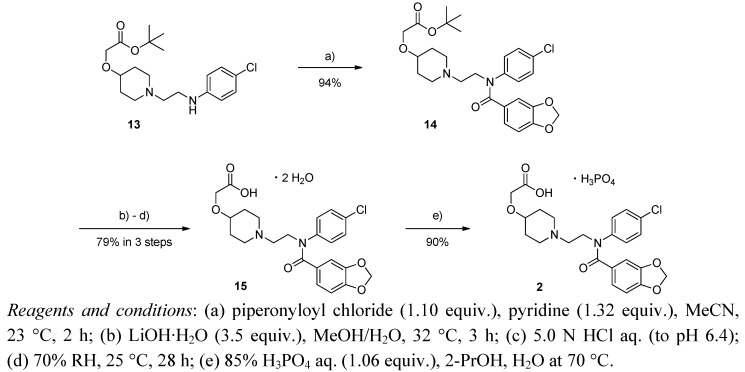
Alternative synthesis of **2**.

Having obtained these successful results, we carried out the final steps of the synthesis of **2** on a 1.00 mol scale, and the synthesis of **2** from **13** was completed as illustrated in Scheme 5. *N*-Acylation of **13** with piperonyloyl chloride in the presence of pyridine as base, hydrolysis of *tert-*butyl ether with excess lithium hydroxide followed by pH adjustment and maintaining conditions of 25 °C and 70% humidity for 28 h furnished **15** as a stable dihydrate. Finally, the formation of its phosphoric acid salt and subsequent recrystallization gave **2** in 90% isolated yield with high purity (>99% by HPLC peak area).

### 2.4. Biological Activity

Competitive binding assays were performed with membrane fractions from CHO or COS-7 cells expressing each human EP receptor subtype. Compound **2** has good selectivity for human IP receptor. For **2**, the K_i_ value for IP receptor was 310 nM and the K_i_ value for other prostanoid receptors was 100-fold the K_i_ value for IP receptor. Compound **2 **also increased cyclic AMP production in COS-7 cells expressing human IP receptor in a concentration-dependent manner, and a significant increase was observed at concentrations of **2** greater than 10 nM. In addition, **2** possesses especially good pharmacokinetic properties. In intravenous administration to dog, the plasma half-life was calculated to be 3.26 h and bioavailability was 86.9%. Further experimental evaluation of the compound’s pharmacological properties is now in progress.

### 2.5. Preparing of Derivatives of 2 as Novel IP Agonists

**Scheme 6:**
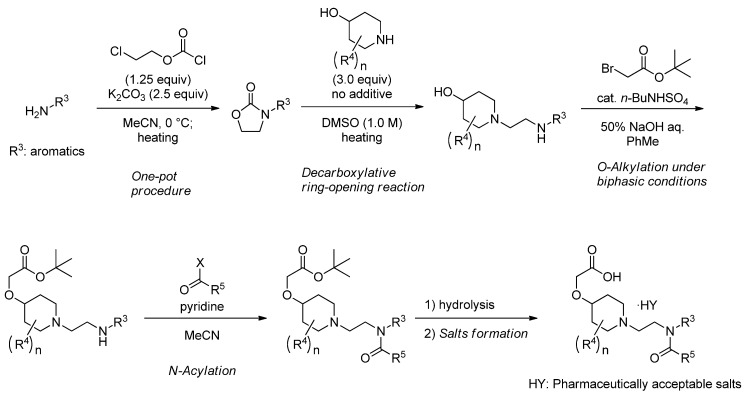
Preparation of derivatives.

The decarboxylative ring-opening reaction enabled preparation of a variety of *N*-aminoethyl cyclic amines derivatives for synthesizing novel IP agonists. Derivatives of **2** were prepared by applying the improved synthetic route consisting of the sequence shown in Scheme 6: (1) one-pot preparation of *N*-aryloxazolidin-2-one; (2) decarboxylative ring-opening reaction; (3) *O*-alkylation under biphasic conditions; (4) *N*-acylation; and (5) salt formation. Structure-activity relationship studies of these novel IP agonists will be published in the future.

## 3. Experimental

### General

NMR [^1^H-NMR (400 MHz)] spectra were determined on a JEOL-LA400 instrument unless otherwise noted. Chemical shifts for ^1^H-NMR are reported in ppm downfield from TMS (d) as the internal standard. Infrared (IR) spectra were recorded on a JEOL-FT/IR-410 and are reported in wavenumbers (cm^−1^). High resolution mass spectra (HRMS) were obtained on a JEOL JMS DX-303 at Toray Research Center, Inc. Elemental analyses were performed with an AT-118 instrument at Toray Research Center, Inc. Melting points (mp), determined on a Yanaco Micro Melting Point Apparatus MP-500, are uncorrected. HPLC analyses were performed on a SHIMAZU LC-10A system with UV detection at a wavelength of 218 nm using YMC-Pack Pro C18 AS-303 (250 × 4.6 mm I.D., S-5 μm). As mobile phase was used a gradient consisting of CH_3_CN and 20 mM H_3_PO_4_-KH_2_PO_4_ buffer (pH = 2.3). All reagents and solvents were of commercial grades and were used without further purification.

*2-Chloro-*N*-(4-chlorophenyl)acetamide.* To a stirred mixture of 4-chloroaniline (600 g, 4.70 mol) in THF (2,126 g) under an Ar atmosphere was added pyridine (391 g, 4.95 mol) at 0 °C. To the mixture was added 2-chloroacetyl chloride (558 g, 4.94 mol) under an Ar atmosphere over 82 min. The reaction mixture was stirred for 30 min at room temperature, and then was cooled to 0 °C. After the reaction was completed, distilled water (1,300 g) and 1 M HCl aq. (1,039 g) were added dropwise to the reaction mixture with cooling at 5 °C. After stirring for 95 min, the generated precipitate was collected by filtration, and then washed with distilled water (1,000 mL). After distilled water (3,000 mL) was added to the resulting precipitate, it was stored at room temperature for overnight. The resulting precipitate was collected by filtration, and washed with distilled water (3,000 mL), and dried to give a title compound (817 g, 85%). Mp 167.9–168.5 °C. IR (KBr): 3501, 3264, 3197, 3130, 3082, 3004, 2952, 2887, 2855, 2795, 2740, 1887, 1765, 1669, 1613, 1551, 1490, 1400, 1340, 1282, 1246, 1189, 1095, 1011, 962, 921, 861, 825, 774, 738 cm^−^^1^. ^1^H-NMR (CDCl_3_): δ = 8.23 (1H, br.s), 7.51 (2H, m), 7.33 (2H, m), 4.19 (2H, s). Elem. Anal. Calcd for C_9_H_10_ClNO: C, 47.09; H, 3.46; N, 6.86; Cl, 34.75. Found: C, 47.09; H, 3.48; N, 6.93; Cl, 34.78.

*4-Chloro-*N*-(2-chloroethyl)aniline hydrochloride.* To a stirred mixture of 2-chloro-*N*-(4-chlorophenyl)acetamide (786 g, 3.85 mol) in THF (6,921 g) under Ar atmosphere was added sodium borohydride (218 g, 5.77 mol) at −7 °C. Then, to the reaction mixture was added boron trifluoride–THF complex (1071 g, 7.65 mol) under Ar atmosphere over 4 h 15 min. The reaction mixture was stirred for 1 h at 67 °C, and then was cooled to 0 °C. Distilled water (803 g) and 50% K_2_CO_3_ aq. (4,997 g) were added dropwise to the reaction mixture at 5 °C. After additional distilled water (2,011 g) and EtOAc (5,440 g) were added, the mixture was stirred for 30 min. The resulting precipitate was separated by filtration. The resulting precipitate was washed with distilled water (899 g) and EtOAc (534 g) respectively. The organic layer was washed with distilled water (2,933 g) and 25% NaCl aq. (2,989 g) respectively. The aqueous layer extracted with EtOAc (2,766 g). Hydrogen chloride in MeOH (1.76 M: 447 g, 1.30 M: 354 g and 2.16 M: 1,332 g) was added dropwise to the combined organic layer at 0 °C. The mixture was stirred for 1 h at room temperature. The mixture was concentrated and residual MeOH was exchanged to EtOAc by evaporation. After EtOAc (3,669 g) was added to the mixture, it was then stirred for 30 min. The resulting precipitate was collected by filtration washed with EtOAc (939 and 934 g), and dried to give a title compound (826 g, 95%). Mp 123.0–124.0 °C. IR (ATR): 2898, 2831, 2766, 2676, 2589, 2455, 2357, 1489, 1435, 1373, 1275, 1266, 1203, 1173, 1099, 1059, 1017, 980, 816, 784, 753 cm^−^^1^. ^1^H-NMR (CD_3_OD): δ = 7.56 (2H, d, *J* = 9.0 Hz), 7.43 (2H, d, *J* = 9.0 Hz), 3.86–3.75 (4H, m). Elem. Anal. Calcd for C_8_H_10_Cl_3_N: C, 42.42; H, 4.45; N, 6.18; Cl, 46.95. Found: C, 42.38; H, 4.40; N, 6.17; Cl, 47.19.

### 3.1. One-Pot Preparation of ***5*** (Scheme 3)

*3-(4-Chlorophenyl)oxazolidin-2-one* (**5**) [21,22]. To a stirred mixture of K_2_CO_3_ (5.32 g, 50.0 mmol) and 4-chloroaniline (2.56 g, 20.0 mmol) in MeCN (40.0 mL) under Ar atmosphere was added 2-chloroethyl chloroformate (2.59 mL, 25.0 mmol) over 5 min at room temperature. After stirring for 1 h, the reaction mixture was heated at reflux for 12 h. The reaction mixture was cooled to room temperature, and then was poured into a mixture of EtOAc (50 mL) and distilled water (75 mL). After the layers were separated, the aqueous layer was extracted with EtOAc (50 mL). The combined organic layer was washed with NaCl aq. (12%, 50 mL), dried over MgSO_4_, filtered and evaporated under reduced pressure to give a crude material. Purifications by recrystallization with EtOAc (32 mL) and *n*-hexane (110 mL) at reflux afforded pure **11** as a solid (6.02 g, 78%). IR (film): 3456, 1735, 1500, 1479, 1425, 1406, 1322, 1219, 1128 cm^−^^1^. ^1^H-NMR (CDCl_3_): δ = 4.05 (dd, *J *= 6.4, 8.0 Hz, 2H), 4.50 (dd, *J *= 6.4, 9.2 Hz, 2H), 7.34 (dt, *J *= 3.2, 10.0 Hz, 2H), 7.34 (dt, *J *= 3.2, 10.0 Hz, 2H).

### 3.2. Optimization of Reaction Conditions (Table 1)

*1-(2-(4-Chlorophenylamino)ethyl)piperidin-4-ol* (**3**) [22]. To a stirred solution of **5 **(163 mg, 1.0 mmol) in DMSO (1.0 mL) under Ar atmosphere was added 4-piperidinol (**4**) (3.0 mmol) at room temperature. The reaction mixture was heated at 110 °C with stirring for 5 days, and then cooled to room temperature. The reaction mixture was partitioned between EtOAc (4 mL) and distilled water (2 mL). After the layers were separated, the organic layer was washed with 6% NaCl aq. (2 mL, three times), dried over Na_2_SO_4_, filtered and evaporated under reduced pressure to give a crude material. Purification by flash column chromatography on silica gel (AcOEt and then 10% MeOH in CHCl_3_) afford **3** (208 mg, 81% yield and 99.0% purity by HPLC peak area) as a light-yellow solid. Mp 84.0–86.0 °C. IR (film): 3364, 3097, 2927, 2830, 1603, 1511, 1474, 1450, 1319, 1265, 1173, 1122, 1067 cm^−1^. ^1^H-NMR (CDCl_3_): δ = 1.48 (br.s, 1H), 1.54–1.63 (m, 2H), 1.89–1.93 (m, 2H), 2.19 (br.t, *J *= 9.6 Hz, 2H), 2.61 (t, *J *= 6.0 Hz, 2H), 2.76–2.79 (m, 2H), 3.12 (dd, *J *= 5.6, 16.8 Hz, 2H), 3.73 (m, 1H), 4.36 (br.s, 1H), 6.55 (dd, *J *= 3.2, 9.6 Hz, 2H), 7.12 (dd, *J *= 3.2, 10.0 Hz, 2H). HRMS (FAB): *m*/*z *[M+H]^+^ calcd for C_13_H_20_ON_2_Cl: 255.1264; found: 255.1260.

### 3.4. *O*-Alkylation of 3 under Biphasic Conditions

tert*-Butyl 2-((1-(2-((4-chlorophenyl)amino)ethyl)piperidin-4-yl)oxy)acetate* (**13**) [21]. To a stirred mixture of **3** (5.00 g,19.6 mmol) in toluene (100 mL) and 50% NaOH aq. (100 mL), tetrabutylammonium hydrogen sulfate (665 mg) as catalyst followed by *tert*-butyl bromoacetate (3.60 mL, 24.4 mmol) were added at room temperature. After the reaction mixture was stirred for 6 h, additional *tert*-butyl bromoacetate (0.73 mL, 4.94 mmol) was added at room temperature. The reaction mixture was additionally stirred for 3 h. Distilled water (100 mL) and toluene (100 mL) were added dropwise to the reaction mixture at room temperature. The mixture was then stirred for 30 min. The layers were separated. The aqueous layer was extracted with toluene (150 mL, three times). The combined organic layer was washed with sat. NaCl aq. (100 mL, three times). The mixture was dried over Na_2_SO_4_, filtered, evaporated to give a residue. To the residue was added EtOH (15.0 mL) at room temperature. The mixture was stirred at reflux, and then was cooled to 0 °C. The resulting precipitate was collected by filtration, and washed with 20 mL of the mixed solvent (*n*-hexane–EtOH = 1:1, v/v), and dried to give **13** (5.78 g, 80%). ^1^H-NMR (CDCl_3_): δ = 7.11 (2 H, d, *J *= 8.4 Hz), 7.74 (2H, d, *J* = 8.4 Hz), 4.35 (1H, br.s), 3.99 (2H, s), 3.43 (1H, ddt, *J* = 4.3, 4.3, 8.5 Hz), 3.10 (2H, dt, *J* = 5.4, 10.8 Hz), 2.76 (2H, dd, *J* = 5.6, 6.5 Hz), 2.59 (2H, t, *J *= 5.9 Hz), 2.17 (2H, br.t, *J* = 9.8 Hz), 1.92 (2H, m), 1.66 (2H, m), 1.48 (9H, s).

### 3.5. Alternative Synthesis of ***2***

tert*-Butyl 2-((1-(2-(N-(4-chlorophenyl)benzo[d][1,3]dioxole-5-carboxamido)ethyl)piperidin-4-yl)oxy)-acetate* (**14**) [20]. To a stirred mixture of **13** (369.3 g, 1.00 mol) in MeCN (1548 g) under Ar atmosphere was added pyridine (104 g, 1.32 mol) at room temperature, and then was cooled to 0 °C. Piperinoyl chloride (203 g, 1.10 mol) in MeCN (785 g) was added dropwise to the reaction mixture over 19 min. The reaction mixture was stirred for 1.5 h at room temperature. To the reaction mixture was added EtOH (275 g), and then was stirred for 2 h at 60 °C. After additional stirring for overnight at room temperature, K_2_CO_3_ (332 g) in distilled water (6,274 g) and EtOAc (3,560 g) were added dropwise to the reaction mixture at room temperature. After the mixture was stirred for 30 min, the layers were separated. The organic layer was washed with 13% NaCl aq. (2,995 g). The mixture was concentrated and residual MeCN was exchanged to EtOAc (1,802 g and 1,790 g) by evaporation. To the resulting precipitate was added EtOAc (1,341 g) with stirring at room temperature. The resulting precipitate was filtered off and washed with EtOAc (478 g). The EtOAc layer was concentrated and residual EtOAc was exchanged to EtOH (942 g and 937 g) by evaporation. To the resulting precipitate was added EtOH (266 g) at room temperature. The mixture was stirred at 70 °C, and then was cooled at 60 °C. After addition of *n*-hexane (728 g), the mixture was stirred for overnight at room temperature. The resulting precipitate was collected by filtration, and washed with 333 g of the mixed solvent (*n*-hexane–EtOH = 9:1, v/v), and dried to give **14** (486 g, 94%), and the purity was 99.6% by HPLC peak area. Mp 111.8–113.8 °C. IR (KBr): 3069, 2957, 2935, 2895, 2863, 2809, 2781, 1739, 1652, 1591, 1577, 1506, 1491, 1443, 1409, 1391, 1368, 1350, 1307, 1295, 1245, 1174, 1159, 1147, 1134, 1116, 1094, 1061, 1039, 1015, 970, 936, 927, 870, 847, 824, 795, 782, 761, 743, 721, 679 cm^−^^1^. ^1^H-NMR (CDCl_3_): δ = 7.20 ppm (2H, d, *J* = 8.5 Hz), 7.03 (2H, d, *J* = 8.5 Hz), 6.79 (2H, d, *J* = 1.5 Hz), 6.77 (1H, dd, *J* = 8.0, 1.5 Hz), 5.92 (2H, s), 3.98 (2H, s), 3.96 (2H, d, *J* = 6.8 Hz), 3.38 (1H, tt, *J *= 8.8, 8.8, 4.4, 4.4 Hz ), 2.77 (2H, dd, *J* = 5.8, 5.4 Hz), 2.54 (2H, t, *J* = 6.7 Hz), 2.15 (2H, br.t, *J* = 9.9 Hz), 1.88 (2H, m), 1.60 (2H, m), 1.48 (9H, s). Elem. Anal. Calcd for C_27_H_33_ClN_2_O_6_: C, 62.72; H, 6.43; N, 5.42; Cl, 6.86. Found: C, 62.82; H, 6.50; N, 5.34; Cl, 6.81.

*2-((1-(2-(*N*-(4-Chlorophenyl)benzo[d][1,3]dioxole-5-carboxamido)ethyl)piperidin-4-yl)oxy)acetic acid* (**15**) [20]. To a stirred mixture of **14** (440 g, 0.851 mol) in MeOH (3,973 g) under an Ar atmosphere was added the solution of lithium hydroxide monohydrate (125 g, 2.98 mol) in distilled water (660 g) at room temperature over 31 min. After the reaction was completed, the pH was adjusted to 6.4 by slow addition of 5.0 M HCl aq. (640 g) over 30 min. The mixture was stored for overnight at room temperature. The mixture was concentrated and residual MeOH was exchanged to distilled water (1,313 g) by evaporation. To the resulting precipitate was added distilled water (1,084 g) at room temperature with stirring. The mixture was stirred at 59 °C for 52 min, and then was cooled to 20 °C. The resulting precipitate was collected by filtration, and washed with distilled water (1,320 g and 1,321 g), and dried to give an anhydrous **15** (319 g, 81%). The anhydrous **15** (310 g, 0.673 mol) was kept at 25 °C under 70% humidity for 28 h to give **15** as a dihydrate (334 g, quant. yield), and the purity was 99.9% by HPLC peak area. Mp 147.3–148.0 °C. IR (KBr): 3367, 2911, 2842, 1644, 1621, 1590, 1504, 1487, 1459, 1441, 1401, 1377, 1311, 1292, 1254, 1218, 1177, 1147, 1113, 1093, 1043, 1012, 968, 953, 941, 930, 898, 870, 834, 817, 761, 740, 719 cm^−^^1^. ^1^H-NMR (DMSO-*d*_6_): δ = 7.34 ppm (2H, d, *J* = 8.7 Hz), 7.19 (2H, d, *J* = 8.7 Hz), 6.79 (1H, d, *J* = 1.5 Hz), 6.76 (1H, d, *J* = 8.0 Hz), 6.72 (1H, dd, *J* = 8.0, 1.5 Hz), 5.99 (2H, s), 3.98 (2H, s), 3.87 (2H, t, *J* = 6.6 Hz), 3.31 (1H, m), 2.64 (2H, m), 2.41 (2H, t, *J* = 6.6 Hz), 2.02 (2H, br.t, *J* = 9.9 Hz), 1.78 (2H, br.d, *J* = 9.8 Hz), 1.36 (2H, m). Elem. Anal. Calcd for C_23_H_25_ClN_2_O_6_·2H_2_O: C, 55.59; H, 5.88; Cl, 7.13; N, 5.64. Found: C, 55.61; H, 5.89; Cl, 7.19; N, 5.59.

*2-((1-(2-(*N*-(4-Chlorophenyl)benzo[d][1,3]dioxole-5-carboxamido)ethyl)piperidin-4-yl)oxy)acetic acid phosphoric acid salt* (**2**) [20]. 2-PrOH (2,819 g) was added dropwise to **15** (280 g, 0.563 mol) in under Ar at room temperature. To the mixture was added phosphoric acid aq. prepared from 85% phosphoric acid (68.5 g) and distilled water (200 g) over 2 min at 70 °C. After addition of a small portion of seed crystal, the mixture was cooled to 20 °C, and then was stirred for overnight at room temperature. The resulting precipitate was collected by filtration, and washed with 2-PrOH (375 g and 373 g), and dried to give **2** (285 g, 90%), and the purity was 99.9% by HPLC peak area. Mp 193.8–195.3 °C. IR (ATR): 1768, 1642, 1489, 1444, 1378, 1332, 1306, 1292, 1260, 1243, 1221, 1157, 1138, 1090, 1069, 1039, 937, 859, 832, 822, 754, 740, 716 cm^−^^1^. ^1^H-NMR (DMSO-d_6_): δ = 7.34 ppm (2H, d, *J* = 8.6 Hz), 7.20 (2H, d, *J* = 8.6 Hz), 6.79 (1H, d, *J *= 1.3 Hz), 6.76 (1H, d, *J* = 8.0 Hz), 6.73 (1H, dd, *J* = 8.0, 1.3 Hz), 5.99 (2H, s), 4.00 (2H, s), 3.91 (2H, t, *J *= 6.7 Hz), 3.36 (1H, m), 2.73 (2H, br.s), 2.53 (2H, m), 2.19 (2H, br.s), 1.81 (2H, br.d, *J *= 10.0 Hz), 1.42 (2H, m). Elem. Anal. Calcd for C_23_H_25_ClN_2_O_6_·H_3_PO_4_: C, 49.43; H, 5.05; Cl, 6.34; N, 5.01. Found: C, 49.46; H, 5.04; Cl, 6.65; N, 4.99.

## 4. Conclusions

In summary, an efficient synthetic method for preparing novel IP agonists was established. This method has the advantage that decarboxylative ring-opening of *N*-aryloxazolidin-2-ones with free cyclic amines in heating DMSO gives *N*-aminoethyl cyclic amines without side reactions. In addition, the subsequent selective *O*-alkylation is suitable for practical industrial scale preparation of **2**. In biological evaluations, **2** exhibited potent and selective IP agonistic activity, and had good pharmacokinetic properties, namely, a long half-life and bioavailability in dog.

## References

[1] Moncada S., Gryglewski R., Bunting S., Vane J.R. (1976). An enzyme isolated from arteries transforms prostaglandin endoperoxides to an unstable substance that inhibits platelet aggregation. Nature.

[2] Collins P.W., Djuric S.W. (1993). Synthesis of therapeutically useful prostaglandin and prostacyclin analogs. Chem. Rev..

[3] Narita S., Takahashi A., Aoki T., Sato H., Satoh S., Yamada S., Kudo M., Yamaguchi T., Kogi K., Shibasaki M. (1993). Syntheses and biological activities of chemically stable prostacyclin mimics with *cis*-bicyclo[4.3.0]nonene ring system: the novel homoisocarbacyclin analogues. Bioorg. Med. Chem..

[4] Skuballa W., Vorbrüggen H. (1981). A new route to 6a-carbacyclins—Synthesis of a stable, biologically potent prostacyclin analogue. Angew. Chem. Int. Ed..

[5] Skuballa W., Schillinger E., Sttirzebecher C.-S., Vorbrüggen H. (1986). Synthesis of a new chemically and metabolically stable prostacyclin analogue with high and long-lasting oral activity. J. Med. Chem..

[6] Ohno K., Nagase H., Matsumoto K., Nishiyama H., Nishio S. (1985). Stereoselective synthesis of 5,6,7-trinor-4,8-inter-m-phenylene-PGI_2_ derivatives and their inhibitory activities to human platelet aggregation. Adv. Prostaglandin Thromboxane Leukot. Res..

[7] Nishio S., Matsuura H., Kanai N., Fukatsu Y., Hirano T., Nishikawa N., Kameoka K., Umetsu T. (1988). The *in vitro* and *ex vivo* antiplatelet effect of TRK-100, a stable prostacyclin analog, in several species. Jpn. J. Pharmacol..

[8] Umetsu T., Murata T., Tanaka Y., Osada E., Nishio S. (1987). Antithrombotic effect of TRK-100, a novel, stable PGI_2_ analogue. Jpn. J. Pharmacol..

[9] Akiba T., Miyazaki M., Toda N. (1986). Vasodilator actions of TRK-100, a new prostaglandin I_2_ analogue. Br. J. Pharmacol..

[10] Miyata M., Ueno Y., Sekine H., Ito O., Sakuma F., Koike H., Nishio S., Nishimaki T., Kasukawa R. (1996). Protective effect of beraprost sodium, a stable prostacyclin analogue, in development of monocrotaline-induced pulmonary hypertension. J. Cardiovasc. Pharmacol..

[11] Kurisu Y., Orihashi K., Sueda T., Kajihara H., Matsuura Y. (1997). Protective effect of beraprost sodium, a stable prostacyclin analogue, on cardiac allograft vasculopathy in rats. Hiroshima J. Med. Sci..

[12] Meanwell N.A., Romine J.L., Seiler S.M. (1994). Non-prostanoid prostacyclin mimetics. Drugs Future.

[13] Wise H., Jones R.L. (1996). Focus on prostacyclin and its novel mimetics. Trends Pharmacol. Sci..

[14] Hayashi K., Nagamatsu T., Oka T., Suzuki Y. (1997). Modulation of anti-glomerular basement membrane nephritis in rats by ONO-1301, a non-prostanoid prostaglandin I_2_ mimetic compound with inhibitory activity against thromboxane A_2_ synthase. Jpn. J. Pharmacol..

[15] Minamoto K. (1997). Beneficial effect of a stable PGI_2_ analogue (ONO-1301) on prostanoid release after reperfusion in canine left single lung allotransplantation model. Nippon Kyobu Geka Gakkai Zasshi.

[16] Kondo K., Hamanaka N. (1995). Prostacyclin mimetics with non-prostanoid structures (in Japanese with English abstract). Folia Pharmacologica Japonica.

[17] Tsubaki K., Taniguchi K., Tabuchi S., Okitsu O., Hattori K., Seki J., Sakane K., Tanaka H. (2000). A novel pyridazinone derivative as a nonprostanoid PGI_2_ agonist. Bioorg. Med. Chem. Lett..

[18] Hattori K., Tabuchi S., Okitsu S., Taniguchi K. (2003). A simple stereoselective synthesis and biological evaluation of FR181157: Orally active prostacyclin mimetic. Bioorg. Med. Chem. Lett..

[19] Nakamura A., Yamada T., Asaki T. (2007). Synthesis and evaluation of *N*-acylsulfonamide and *N*-acylsulfonylurea prodrugs of a prostacyclin receptor agonist. Bioorg. Med. Chem. Lett..

[20] Hayashi, R.; Sakagami, H.; Koiwa, M.; Makita, K. Cyclic amines and their use for blood platelet aggregation inhibitory pharmaceuticals. *Jpn. Kokai Tokkyo Koho* JP 106692, 2007.

[21] Morita, Y.; Kawamura, K. Preparation of *N*-phenylethylenediamine derivatives. *Jpn. Kokai Tokkyo Koho *JP 197397, 2007.

[22] Morita Y., Ishigaki T., Kawamura K., Iseki K. (2007). Short and practical synthesis of *N',N'*-disubstituted *N*-aryl-1,2-ethylenediamines by a decarboxylative ring-opening reaction under nucleophilic conditions. Synthesis.

[23] Dermer O.C., Ham G.E. (1969). Ethylenimine and other Aziridines.

[24] Meléndez R.E., Lubell W.D. (2003). Synthesis and reactivity of cyclic sulfamidites and sulfamidates. Tetrahedron.

[25] Poindexter G.S. (1983). The use of 2-oxazolidinone as a latent aziridine equivalent. I. A facile method for the preparation of 2-substituted oxazolines. J. Heterocycl. Chem..

[26] Poindexter G.S. (1983). Aminoethylation.

[27] Poindexter G.S., Owens D.A., Dolan P.L., Woo E. (1992). The use of 2-oxazolidinones as latent aziridine equivalents. 2. Aminoethylation of aromatic amines, phenols, and thiophenols. J. Org. Chem..

[28] Rooney P.C., Nutt M.O. (1996). Aminoethylation process for production of substituted ethylenediamines involving oxazolidinone ring opening with secondary amines or alkanolamines.

[29] Hata Y., Watanabe M., Shiratori O., Takase S. (1978). Cytotoxic activity and fragmentation of aziridines in microsomes. Biochem. Biophys. Res. Commun..

[30] Fishbein L. (1980). Potential carcinogenic and mutagenic industrial chemicals, I. Alkylating agents. J. Toxicol. Environ. Health.

[B31-molecules-17-01233] 31.Available anilines were converted to *N*-substituted chloroethylaniline hydrochloride salts via a three-step sequence that included *N*-acylation, selective reduction of the carbonyl group with BH_3_-THF, and hydrochloride salt formation (the details of synthesis of 4-chloro-*N*-(2-chloroethyl)aniline hydrochloride are described in “3. Experimental Section”).

[32] Sørensen J.K., Fock J., Pedersen A.H., Petersen A.B., Jennum K., Bechgaard K., Kilsa K., Geskin V., Cornil J., Bjørnholm T. (2011). Fulleropyrrolidine end-capped molecular wires for molecular electronics; Synthesis, spectroscopic, electrochemical, and theoretical characterization. J. Org. Chem..

[33] Rudesill J.T., Severson R.F., Pomonis J.G. (1971). The syntheses of *N*-arylaziridines. J. Org. Chem..

[35] Yin J., Buchwald S.L. (2002). Pd-Catalyzed intermolecular amidation of aryl halides: The discovery that Xantphos can be *trans*-chelating in a Palladium complex. J. Am. Chem. Soc..

[36] Mallesham B., Rajesh B.M., Rajamohan R., Srinivas D., Trehan S. (2003). Highly efficient CuI-catalyzed coupling of aryl bromides with oxazolidinones using Buchwald’s protocol: A short route to Linezolid and Toloxatone. Org. Lett..

[37] Ghosh A., Sieser J.E., Caron S., Couturier M., Dupont-Gaudet K., Girardin M. (2006). Cu-Catalyzed *N*-arylation of oxazolidinones: An efficient synthesis of the *k*-opioid receptor agonist CJ-15161. J. Org. Chem..

